# Recent developments in synthetic biology and metabolic engineering in microalgae towards biofuel production

**DOI:** 10.1186/s13068-018-1181-1

**Published:** 2018-06-30

**Authors:** Sheeja Jagadevan, Avik Banerjee, Chiranjib Banerjee, Chandan Guria, Rameshwar Tiwari, Mehak Baweja, Pratyoosh Shukla

**Affiliations:** 1 Department of Environmental Science and Engineering, Indian Institute of Technology (Indian School of Mines), Dhanbad, Jharkhand 826004 India; 2 Enzyme Technology and Protein Bioinformatics Laboratory, Department of Microbiology, Maharshi Dayanand University, Rohtak, Haryana 124001 India; 3 Enzyme and Microbial Biochemistry Lab, Department of Chemistry, Indian Institute of Technology, Hauz-Khas, New Delhi 110016 India

**Keywords:** Synthetic biology, Microalgae, Biofuel, Optimization models, Genome-scale reconstruction, Biorefinery

## Abstract

In the wake of the uprising global energy crisis, microalgae have emerged as an alternate feedstock for biofuel production. In addition, microalgae bear immense potential as bio-cell factories in terms of producing key chemicals, recombinant proteins, enzymes, lipid, hydrogen and alcohol. Abstraction of such high-value products (algal biorefinery approach) facilitates to make microalgae-based renewable energy an economically viable option. Synthetic biology is an emerging field that harmoniously blends science and engineering to help design and construct novel biological systems, with an aim to achieve rationally formulated objectives. However, resources and tools used for such nuclear manipulation, construction of synthetic gene network and genome-scale reconstruction of microalgae are limited. Herein, we present recent developments in the upcoming field of microalgae employed as a model system for synthetic biology applications and highlight the importance of genome-scale reconstruction models and kinetic models, to maximize the metabolic output by understanding the intricacies of algal growth. This review also examines the role played by microalgae as biorefineries, microalgal culture conditions and various operating parameters that need to be optimized to yield biofuel that can be economically competitive with fossil fuels.
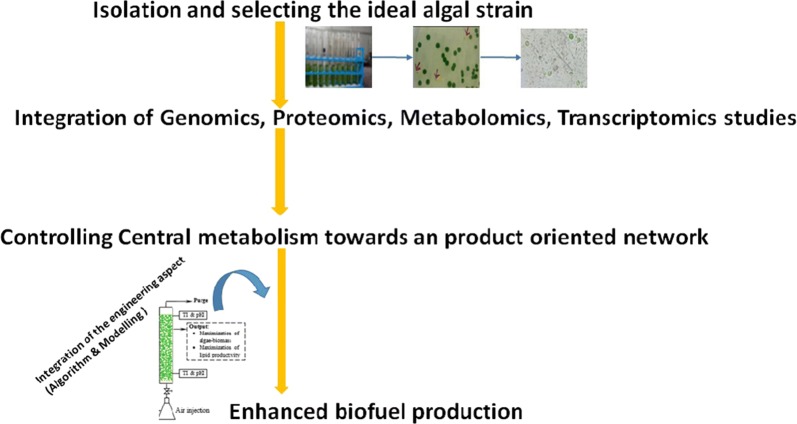

## Background

An upsurge in global population coupled with rapid industrialization has tremendously increased worldwide per capita energy consumption. This rising energy demand is being fulfilled by various non-renewable energy sources, particularly fossil fuels. It is estimated that at the present usage, the worldwide demand for energy is envisaged to escalate by more than 50% by the year 2030 [1]. With a concomitant addition of 1.7 billion in the global population by 2050, the dwindling fossil fuel reserves will be subjected to further pressure [2]. The energy consumption rate for non-renewable sources such as natural petroleum is reported to be 105 times faster than the replenishment rate, which calls for an immediate attention to look for alternative, renewable and sustainable energy resources [1].

Biomass-derived biofuels are currently being considered as the most sustainable alternative to fossil fuels. Herein, problems such as water pollution and carbon dioxide emission associated with combustion of fossil fuels can be mitigated. Biofuels have been segregated into four generations based on their feedstock material [3–5]. Various cereals, sugarcane and oil-containing food crops constituted the feedstock for the first-generation biofuels. Sugar and starch-containing plants or cereal crops were used in the production of first-generation bioethanol. Production of bioethanol and methanol from low-cost crops, agricultural and forest residues (trees, straw, bagasse) constitutes the second-generation biofuel. The increased competition of crop-based first- and second-generation biofuels with food crops for arable land coupled with high corrosivity and hygroscopicity of bioethanol does not portray it to be an ideal candidate for replacement of fossil fuels. The third-generation biofuels exploit marine biomass such as seaweeds and algae for the generation of biofuels such as biogas, ethanol and butanol. Recent developments in synthetic and systems biology are paving the way for the fourth-generation biofuels, wherein specially engineered microorganism/crops will serve as feedstock material. Researchers are therefore, actively involved in developing alternative biofuel molecules, with structures similar to short-chain, branched-chain and cyclic alcohols, alkanes, alkenes, esters and aromatics.

**Fig. 1:**
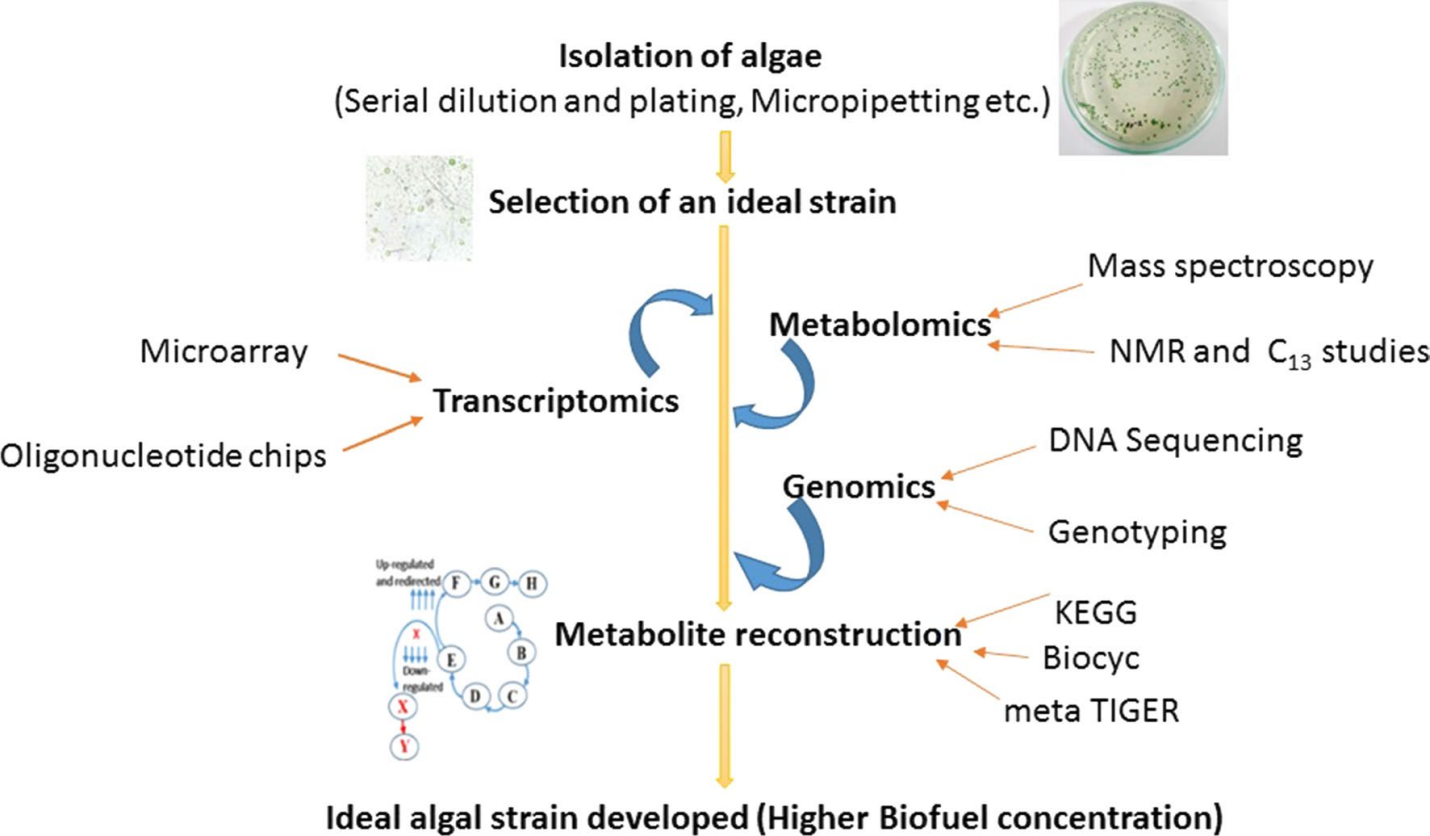
Pictorial representation of the overall process towards biofuel production in microalgae using synthetic biology approach (i.e., isolation, selection of an ideal strain, redirecting the metabolism to maximize synthesis of the targeted biofuel)

Amongst various biomass, microalgae are gaining much attention for use as a potential feedstock by virtue of its high carbohydrate and lipid content, rapid growth rate and resistance to fluctuating environmental conditions [6]. Advancement in reverse engineering tools such as genome sequence, genetic transformation, gene targeting, different promoters, selection markers and molecular biology techniques such as clustered regularly interspaced short palindromic repeats (CRISPR), transcription activator-like effectors (TALEs) and zinc-finger nucleases (ZFN) have paved the way to unravel novel metabolic pathways occurring within the algal cells or to design and synthesize new biological systems, all of which are aimed at furthering biofuel production [7–10]. Figure 1 illustrates the overall process involved in microalgae-based biofuel production. Recent studies conducted on model microalgae such as *Chlamydomonas reinhardtii* [11–15], *Chlorella pyrenoidosa* [16], *Neodesmus* sp. UTEX 2219-4 [17], *Scenedesmus* [18], *Phaeodactylum tricornutum* [19, 20], *Dunaliella salina* [21], *Dunaliella parva* [22], *Nannochloropsis oceanic* [23], *Nannochloropsis salina* [24], cyanobacteria [25] compliments the advances in molecular biology tools and facilitates to construct novel biological systems via synthetic biology. The present review deals with the recent developments in algal biorefinery, synthetic biology, metabolic engineering tools and the optimization of algal culture conditions through an algorithm, to address pressing issues related to algal biofuel production. This review also examines the role played by microalgae as biorefineries, microalgal culture conditions and various operating parameters that need to be optimized to yield biofuel that can be economically competitive with fossil fuels.

## Products from algal biorefinery

Development of microalgae-based biofuels in itself is not an economically competitive alternative to existing technologies and hence focus has now shifted towards abstraction of high-value co-products from microalgae to improve the economics of microalgae-based biorefinery. Biorefinery approach is a system where energy, fuel, chemicals and high-value products such as pigments, proteins, lipids, carbohydrates, vitamins and antioxidants are produced from biomass through various processes. Microalgae are rich in proteins, lipids and carbohydrates and the relative amounts of these biochemical components vary amongst various microalgal strains [26]. These could be used as feedstock for the production of various high-value bio-based products such as biodiesel production from microalgal lipids, alternate carbon source in the fermentation industries of microalgal carbohydrates, health food supplements from long-chain fatty acids found in microalgae and in pharmaceutical applications [27]. Recent studies conducted on microalgae for the production of various biofuels are listed in Table 1.

**Table 1:** Recent studies in microalgae employed for the production of biofuel

S. no.	Algal species	Reactor/growth conditions and products	References
1	*Chlamydomonas reinhardtii* GY-D55	Flat-plate airlift photobioreactor. Production rate 0.000959 kg H_2_/kg dry cells/h	[142]
2	*Nannochloropsis gaditana*	Down-flow type supercritical water gasification reactor. Product composed of hydrogen (52.0%), methane (17.9%) and CO_2_ (23.0%) with 97.4 wt% gasification efficiency	[143]
3	*Chlorella vulgaris*	Supercritical water gasification of microalgal hydrothermal liquefaction for hydrogen production. Non-stirred batch stainless steel Parr reactor produced 30 mol H_2_/kg algae	[144]
4	*Laminaria digitate*, *Chlorella pyrenoidosa*, *Nannochloropsis oceanica*	Two-stage batch co-fermentation resulted in 6.6 and 70.9% energy conversion efficiencies during hydrogen fermentation and combined H_2_–CH_4_ production, respectively. Hydrogen yield of 94.5–97.0 mL/g volatile solids	[145]
5	*Chlamydomonas* sp., *Chlorella* sp., *Chlorella vulgaris TISTR 8580* and *Chlorella protothecoides*	Biophotolysis-based hydrogen and lipid production using crude glycerol as an exogenous carbon source. The optimal conditions were glycerol concentration of 16 g/L, initial pH 6.8, and light intensity of 48 μmol photon/m^2^ s, yielding 11.65 ± 0.65 mL/L hydrogen along with lipid content > 40% in the microalgal biomass	[146]
6	*Scenedesmus dimorphus*	Combination of separate hydrolysis and fermentation and simultaneous saccharification and fermentation found most effective. Lipid-extracted biomass yielded 0.26 g bioethanol/g lipid-extracted biomass at pH 5, temperature of 34 °C, and microalgae biomass loading at 18 g/L	[147]
7	*Dunaliella* sp.	Microalgae used as a feedstock for bioethanol production. 72 h of incubation at substrate concentration of 30 g/L microalgal biomass and 3 mL inoculums at pH 6 yielded 7.26 g/L bioethanol	[148]
8	*Scenedesmus dimorphus*	Microalgae used as a feedstock for bioethanol production. Fermentation conducted at pH 5, temperature of 34 °C, and microalgae biomass loading at 18 g/L via simultaneous saccharification and fermentation resulted in a theoretical yield of bioethanol that exceeded 90%	[149]
9	Mixed microalgae cultures	Fermentation of the glucose after enzymatic hydrolysis yielded 0.46 g ethanol/g glucose	[150]
10	*Porphyridium cruentum*	Freshwater biomass produced ethanol more efficiently than the sea water biomass with ethanol conversion yields of 70.3 and 65.4%, respectively, after 9 h. Simultaneous saccharification and fermentation processing was superior to separate hydrolysis and fermentation processing for bioethanol production	[31]
11	*Scenedesmus acutus*	Purified lipids were catalytically deoxygenated to yield liquid product consisting of 99 wt% hydrocarbons and diesel-like (C10–C20) hydrocarbons	[151]
12	*Dunaliella tertiolecta*	Carbon nanotube (CNT)-supported metal catalyst for hydrothermal liquefaction of *Dunaliella tertiolecta* to produce bio-oil. Bio-oil conversion and yield increased to 95.78 and 40.25 wt%, respectively, when Co/CNTs were employed as catalysts	[13]
13	*Euglena sanguinea*	Catalyst calcinated from natural white mussel shell at 1000 °C used in the transesterification process. The algal biodiesel showed the presence of saturated fatty acids: C16:0, C18:0, C22:0, C24:0 and monounsaturated fatty acids C18:1	[41]

Variety of algal strains such as green alga *C. reinhardtii*, cyanobacteria *Anabaena cylindrica* and *A. variabilis* are well known for hydrogen production in the presence of sunlight. The aforementioned organisms are capable to extract and direct protons and electrons derived from water to hydrogen production, catalyzed via chloroplast hydrogenases, namely HydA1 and A2 (Hydrogenase) [28, 29]. Kruse et al. [28] observed that the native bio-hydrogen production rate in *C. reinhardtii* was improved (maximal rate of 4 mL/h) by inducing modification in its respiratory metabolism by eliminating potential competition for an electron with hydrogenase. Similarly, heterologous expression of hexose uptake protein (HUP1 hexose symporter from *Chlorella kessleri*) in a hydrogen-producing mutant (Stm6) of *C. reinhardtii* revealed approximately 150% increase in H_2_ production capacity [30]. With increased research focusing on third-generation biofuels, several recent studies on bioethanol production by employing algal strains such as *Porphyridium cruentum* [31], *Tetraselmis suecica* [32] and *Desmodesmus* sp. [33] have been reported. An algal strain *Synechocystis* sp. PCC 6803 produced via double homologous recombination system was capable of photoautotrophically converting CO_2_ to bioethanol [34], with a maximum theoretical yield of 0.696 g ethanol/g CO_2_, as against 0.51 g ethanol/g glucose by *S. cerevisiae.* Active research needs to be directed in the field of algae-based bioethanol production, focusing on improvement in the yield to make the process economically viable.

Several recent studies indicated the production of bio-butanol from various microalgae such as carbohydrate-rich microalgae *Neochloris aquatica* CL-M1 [35], acid-pretreated biomass of *Chlorella vulgaris* JSC-6 [14] and microalgae-based biodiesel residues [36]. An algal strain, *Synechococcus elongates*, was reported to produce butanol synchronized with the production of isobutyraldehyde and isobutanol [37]. The genetically engineered *S. elongates* was able to produce isobutyraldehyde at a higher rate than those reported for ethanol, hydrogen or lipid production by either cyanobacteria or algae by the upregulation of ribulose bisphosphate carboxylase/oxygenase. The major obstacle in large-scale commercialization of microalgae-based bio-butanol is, however, the generation of several byproducts which reduces the purity and yield of pure butanol.

Biodiesel has emerged as a promising alternative for fossil fuels by virtue of having similar chemical characteristics with the latter. Most microalgae act as a resource for large-scale production of biodiesel owing to the high biomass productivity coupled with the rapid accumulation of lipid. Diverse algal genera such as *Nannochloropsis*, *Dunaliella*, *Chaetoceros*, *Botryococcus*, *Scenedesmus* and *Pseudochlorococcum* are known to accumulate high amount of neutral lipids [38–40]. The metabolic pathways of algal strains are able to produce 16–20 carbon fatty acids as precursors for the production of biodiesel. Nobre et al. [38] employed *Nannochloropsis* sp. for the production of fatty acid for biodiesel production and other co-products such as carotenoids and bio-hydrogen. This study reported that when CO_2_ supercritical fluid extraction was used, 45 g/100 g dry biomass of lipids and 70% of pigments were extracted. In a recent study, a high-lipid-producing microalga, namely *Euglena sanguinea* was investigated for biodiesel production [41]. The saturated fatty acids (C16:0, C18:0, C22:0, C24:0) and unsaturated fatty acids (C18:1) in the biodiesel confirmed that these could be potentially used in automobiles without any considerable transition in engine design. Here, systems biology, especially flux analysis, can provide an effective means of prediction by tracking the carbon flux during lipid accumulation, carbon fixation and growth altogether. The enzymes that can be targeted to enhance growth and carbon fixation can be determined from enzyme flux control coefficient data of Calvin cycle enzymes [42]. Likewise, the targets involved in lipid metabolism can also be found by 13C metabolic flux data and subsequent metabolic map derived from oleaginous algae. These flux data reveal which enzymes and the pathways they regulate are rate limiting and exert significant control over the larger metabolism [43, 44]. Nowadays, researchers are focusing on metabolic engineering pathways aimed at enhancing the fatty acid chain length, overexpression of genes involved in fatty acid synthesis with simultaneous down-regulation of genes involved in β-oxidation and lipase hydrolysis. These developments would facilitate to increase the yield, concurrent with an economical algal biodiesel production in the near future.

In addition to biofuels, microalgae are feedstock to several other high-value products such as vitamins, pigments, proteins, carbohydrates, amino acids, antioxidants, high-value long-chain polyunsaturated fatty acids (PUFAs) and biofertilizers. Natural microalgal pigments such as carotenoids, chlorophylls and phycobiliproteins serve as precursors of vitamins in food, pharmaceutical industries, cosmetics and coloring agents [45]. Several studies are focussing on microalgal genes encoding enzymes, which are involved in high-value carotenoid synthesis. Microalgae such as *C. reinhardtii* do not naturally synthesize ketocarotenoid, and β-carotene ketolase gene from *Haematococcus pluvialis* was expressed in *C. reinhardtii* to synthesize a new ketocarotenoid [46]. In another study, *C. vulgaris* was transformed with promoter and terminator of the nitrate reductase gene from *P. tricornutum* and the transgenic strain proved valuable for the production of value-added proteins [47]. Microalgae also serve as potential expression systems for the synthesis of biopolymers such as poly-3-hydroxybutyrate, which is a key precursor for the synthesis of biodegradable plastics. In a recent study, an ATP hydrolysis-based driving force module was engineered into *Synechococcus elongatus* PCC 7942 to produce 3-hydroxybutyrate [48]. The strain which was engineered by having a provision for a reversible outlet for excessive carbon flux was capable of producing significantly high amounts of 3-hydroxybutyrate over the native strain. Several microalgae secrete extracellular polymeric substances in their immediate living environment as a hydrated biofilm protective matrix [49]. These substances are known for high-value applications such as anti-inflammatories, antivirals, antioxidants, anticoagulants, biolubricants and drag reducers.

Spent microalgal biomass or lipid-extracted algal (LEA) biomass is an attractive feedstock for the production of various products as it contains 30–60% of residual carbon that is in the form of readily fermentable sugar [50]. In a recent study, LEA has been used as a substrate for biomethanation through anaerobic processes [51]. This study showed that the rate of biogas production was comparatively higher in product-extracted algal samples (lipid and protein extracted), whilst the cumulative methane production was higher for pretreated algae (dried powdered algae and heat-treated algae). A related study conducted by Hernández et al. [52] reported that anaerobic digestion of lipid-exhausted biomass showed higher yields of methane than non-lipid-exhausted biomass. LEA has also been used as raw material for butanol fermentation [53]. Bench-scale tests demonstrated that LEA could also be effectively converted to liquid fuel, mainly alkanes via hydrothermal liquefaction and upgrading processes such as via hydrotreating and hydrocracking. The overall energy efficiency on a higher heating value basis of this process was estimated to be 69.5% [54]. A study conducted by Gu et al. [55] demonstrated that *Scenedesmusacutus* was found to be capable of assimilating nitrogen from LEA residuals and was able to replace nitrate in culturing media, thus facilitating nutrient recycling. Another recent study reports that nitrogen/phosphorus limitation in microalgae leads to variation in lipid productivity, which was validated by the expression of acetyl-CoA carboxylase gene [25].

## Bioprocesses for algal cultivation

Photosynthesis-dependent accumulation of biomass takes place under nitrogen-rich environment, whereas accumulation of lipids in microalgae occurs under stress conditions such as limited nutrient conditions. This contradiction hugely offsets continuous lipid production, thus affecting the economics of the system. The fed batch process has often been reported as the most suitable method for microalgal cultivation, because it offers the flexibility of customization of nutrients provided during the process. A study conducted by Zheng et al. [56] demonstrated that during the fed batch process, the lipid content and algal biomass were markedly increased. Moreover, studies proved that supply of light in phototrophic and mixotrophic processes during the fed batch culture increased the lipid productivity [57]. Light spectral quality also plays a key role in facilitating photosynthesis. Absorption of irradiation corresponding to the absorption band of the algal chlorophyll can lead to enhanced photosynthesis [58]. In a recent study, batch process was attempted in an open thin-layer cascade photobioreactor for high-cell density cultivation of a saline microalga (*N. salina)*, wherein maximal cell density of 50 g/L was obtained within 25 days [59]. Similarly, fed batch heterotrophic microalgae cultivation of *Auxenochlorella protothecoides* was employed to maximize lipid production. An optimal feeding strategy was determined by interior point optimization [60]. On the contrary, Tang et al. [61] reported that long-term steady-state continuous process leads to significant lipid accumulation since the rate of dilution and irradiance can be regulated at specific levels. Furthermore, to maintain the steady state, the system is continuously being supplied with essential nutrients, which prevents the formation of inhibitory metabolites.

Although continuous feeding is required for maintenance of growth rate, the accompanying non-limiting nutrient supply may hamper accumulation of lipid and carbohydrates. To maintain a balance between lipid production and algal biomass production, a two-stage cultivation process was applied, wherein different set of conditions were supplied for growth and high lipid content/carbohydrate [62]. Thereafter, studies proved that semi-continuous strategy is most beneficial for algal biomass and lipid accumulation for longer periods because of the persistence of culture at exponential phase [63]. A recent study reported a two-stage continuous cultivation of *Chlorella* sp., where process variables such as dilution rate and feed stream substrate concentrations were optimized for biomass productivity and continuous production of lipid-rich algal biomass was monitored in two sequential bioreactors [64].

Under heterotrophic growth conditions, microalgae utilize a source of organic carbon as substrate for growth, which is converted to lipids. Due to the dependency on the availability of a continuous source of sugar, the cost of the process increases considerably. To overcome the aforementioned hurdles, microalgae are grown mixotrophically. Microalgae such as *Chlorella* sp. and *Nannochloropsis* sp. have been grown mixotrophically for consideration for biodiesel production [57]. In a comparative study between autotrophic, heterotrophic and mixotrophic cultivation, it was demonstrated that strains of microalgae *Tribonema* sp. showed enhanced growth in heterotrophic conditions, whereas lipid production was maximum during mixotrophic growth conditions [65]. Though several studies are focusing on these aspects, an effective microalgae-based system which does not compromise on growth or lipid content has not yet been developed.

## Microalgae-based biofuel engineering

**Fig. 2:**
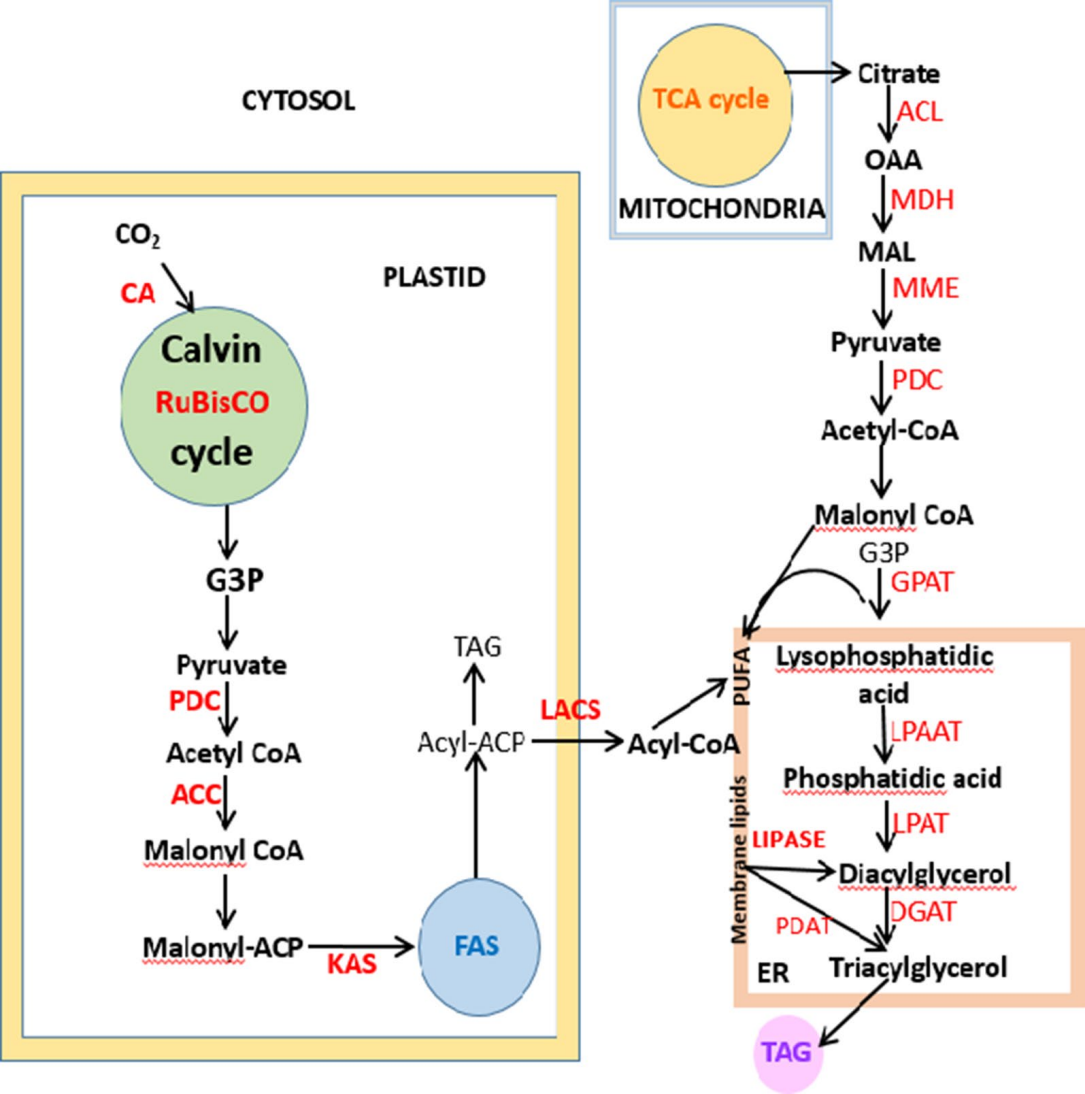
Scheme representing the synergy between enzymes that lead to the formation of lipid (*CA* carbonic anhydrase; *RuBisCO* Ru1,5BP carboxylase/oxygenase; *PDC* pyruvate dehydrogenase complex; *ACC* acetyl-CoA carboxylase; *KAS* 3-ketoacyl-ACP synthase; *ACL* ATP-citrate lyase; *MDH* malate dehydrogenase; *MME* NADP-malic enzyme; *PDC* pyruvate dehydrogenase complex; *GPAT* glycerol-3-phosphate acyltransferase; *LPAAT* lyso-phosphatidic acid acyltransferase; *LPAT* lyso-phosphatidylcholine acyltransferase; *DGAT* diacylglycerol acyltransferase; *PDAT* phospholipid diacylglycerol acyltransferase

In addition to maintaining the right balance between the biomass content and lipid production, microalgae-derived biofuel faces several other challenges such as inefficient harvesting techniques and low productivity due to ineffective photobioreactor design and limited photosynthetic efficiency [66]. Genetic engineering tools aimed at overexpression of enzymes which are involved in lipid synthesis and storage and/or reduction of lipid catabolism mechanisms, offer a huge potential to engineer the required metabolisms. Figure 2 illustrates the interactions amongst various enzymes involved in the synthesis of lipids by algae. Over the years, research has been focused on overexpression of acetyl-CoA carboxylase (ACCase) involved in fatty acid synthesis and knocking out of genes involved in fatty acid oxidation (acyl CoA oxidase, acyl CoA synthase, carnitineacyltransferase I, fatty acyl CoA dehydrogenase) [17, 67]. Genetic manipulation in microalgae is challenging as editing tools are species specific and cannot be used interchangeably because of codon usage, defensive strategies (viz. restriction enzymes and methylation), uptake of nucleic acid and porosity of cells. Therefore, an extensive knowledge of the genetic construction of individual species is a prerequisite for customization of microalga for target biofuel production [68]. At present, there are a limited number of fully annotated genomes of microalgae available, but with improvements in sequencing techniques, rapid increase in such information is foreseeable in the near future.

### Synthetic biology in microalgae

The crux of synthetic biology is to be able to build artificial regulatory circuits that can control cellular behavior based on user-defined inputs/signals/stimuli to generate the desired output such as biofuel chemicals and proteins by making required changes in metabolism. The main focus of microalgal biotechnology for large-scale application of microalgae as a sustainable and robust energy feedstock is based on (a) improving their photosynthetic efficiency through metabolic engineering for improved oil yield and enhanced carbon sequestration rate in mass cultures, (b) driving the carbon flux into energy-rich compounds useful as biofuel source and (c) development of robust and committed algal cells that can sustain low-cost large-scale cultivation resulting in lower operational costs and lower carbon footprint of the produced chemical [69]. Synthetic biology functions by the construction of artificial biological systems by an amalgamation of engineering strategy with biotechnological tools, based on the genetic, metabolic and regulatory data gathered from experiments [69, 70]. In simple words, it is the field of biology which focuses on reconstruction or re-engineering the metabolic pathways with the introduction of genetic modules and which are observed as “biological circuits’’. The objective is to reprogram microalgae to derive a novel function. The reconstruction of relevant biochemical pathways (that yield metabolite of importance) into partial or total synthetic alternatives needs a stable assembly along with the integration of heterologous DNA segments into hosts or chassis strains. The various ways genes can be fabricated for reconstructions are (a) ligation and digestion of DNA fragments, (b) in vitro homologous recombination of fragments and (c) in vivo homologous recombination [59].

#### Elements of synthetic circuits/biobricks

In a bid to commercialize transgenic microalgae, certain challenges need to be addressed. These are primarily poorly developed molecular tools for genetic engineering and low level of heterologous gene expression from the nucleus [69, 71]. With the advent of novel genomic tools, synthetic biology is fast developing and the concept of “BioBricks” could be introduced in microalgal systems. These biobricks are standardized DNA segments having common interface, which can be assembled into biological systems. Such parts are interchangeable units such as promoters [72–74], terminators, ribosome-binding sites (RBS), trans-elements and various regulatory molecules that can be used to regulate genetics of microalgae and ultimately their metabolism [75]. In addition to the aforementioned genetic tools, “omics” approach could play a vital role in structuring pathway reconstruction in microalgae by helping us understand the metabolism in the whole system, which is regulated through feedback-forward and -backward loops that affect the output. The huge amount of available ‘omics’ data could also be channeled towards attaining lipid-inducing conditions (i.e., stress stimulus), thus leading to an efficient regulation of metabolism via application of synthetic biology by targeting the regulatory networks rather than mere deletion or overexpression of enzyme-coding genes [76–79].

#### Microalgal context

**Fig. 3:**
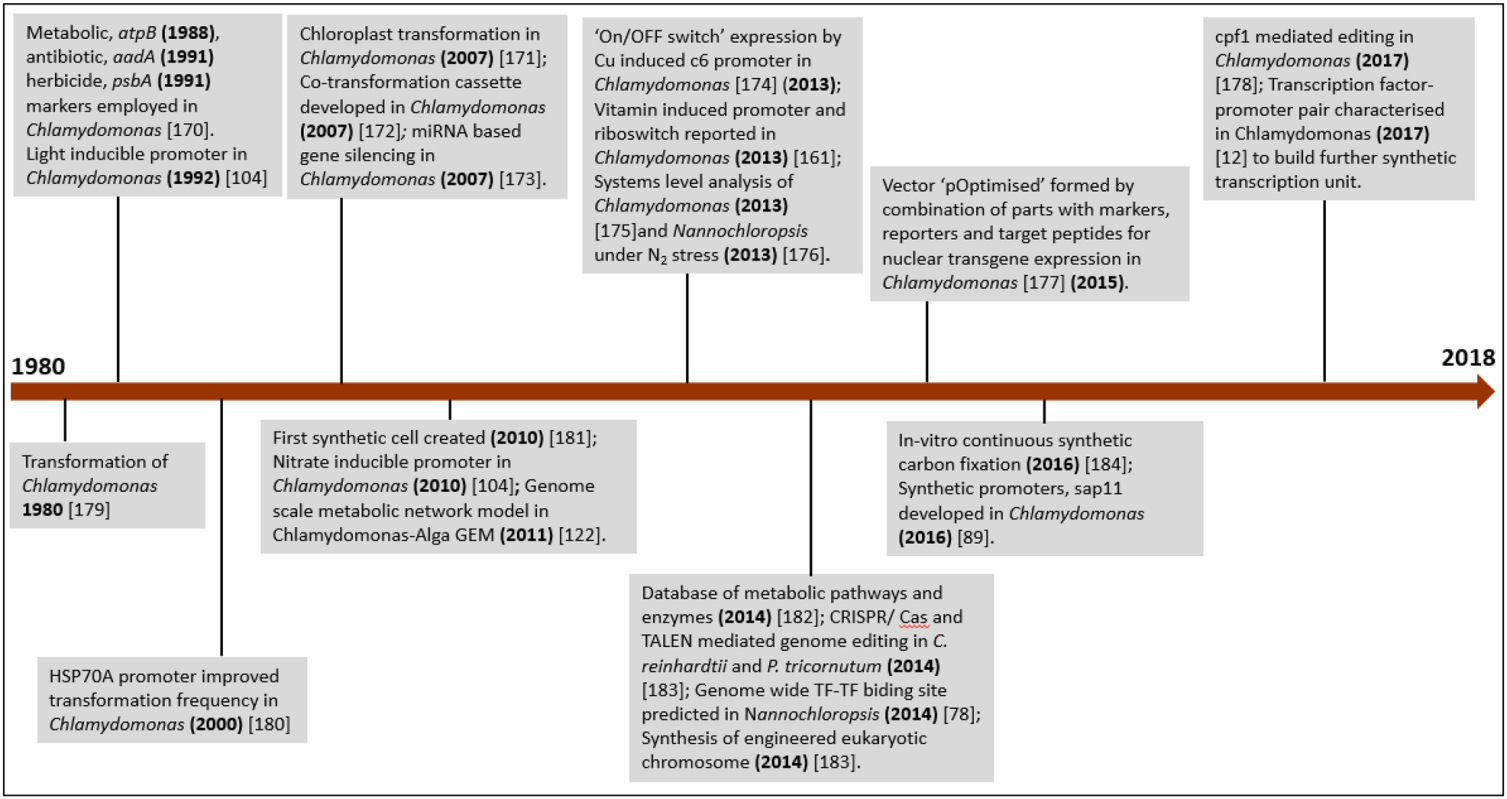
Key events that mark the development of synthetic biology in microalgae-based oil accumulation

Synthetic biology could be presently applied to systems with reverse genetic tools and have developed resources such as genomic sequence, selection markers, plasmid vectors, various promoters and genetic editing tools. However, these are presently being limited to selected microalgal species. The microalgal models that can be congenial to reverse genetics and synthetic biology perturbations based on the existing genetic resources are available for *C. reinhardtii*, *Nannochloropsis* sp., *P. tricornutum*, *Cyanidioschyzon merolae*, *Ostreococcus tauri* and *Thalassiosira pseudonana* [70]. Traditionally, most of the applications of synthetic biology have been carried out in a model microalga, *C. reinhardtii*, where existing tools are being modified and optimized, synchronous with the development of novel tools. Therefore, in *C. reinhardtii*, protein production has been accomplished by engineering the chloroplast genome because reverse genetics and editing methods could be attempted in chloroplasts. With the development of expression constructs [80, 81], protein production has been recorded to reach 80 times more in chloroplasts than that of nuclear expression [82, 83]. Attempts have been made to alter the algal photosynthetic machinery by synthetic biology via expression of heterologous genes (gene *psbA* that codes D1 subunit of Photo system II) in chloroplasts as “Biobricks” under the influence of endogenous regulatory system [84]. Efforts have also been carried out to engineer Rubisco under varying external environment, to enable optimized utilization of energy and carbon. This was carried out by altering the amount of *rbcl* mRNA (large subunit genes of Arabidopsis Rubsico) maturation factor MRL1 from the nuclear genome of MRL1-deficient mutant. Rubisco content has been reported to reduce by 15% in comparison with the wild type, with no reduction observed in their growth. Interestingly, in the presence of inducible promoters, Rubisco amount could be altered under varying light intensity and CO_2_ concentration, as shown by Johnson [85]. Key events that mark the development of synthetic biology in microalgae-based oil accumulation are illustrated in Fig. 3.

Lipid metabolism is highly complex and tightly regulated with an elaborate regulatory network of nodes and internodes. Hence, the characterization of pathways with an extensive knowledge about carbon sharing is imperative for fabrication of artificial pathways. Investigations have been carried out to observe carbon partitioning between starch and lipid and attempts have been made to isolate the key players of triacylglycerol (TAG) formation under stress in *C. reinhardtii* [86]. Lipid engineering has been carried out in microalgae with an aim to produce TAG with better cold flow properties, making them a better feedstock amenable for biodiesel production. In diatom *P. tricornutum*, the ratio of short-chain fatty acids C12 and C14 were increased by enhanced expression of thioesterases derived from different plants [87]. Similar efforts have been carried out in *C. reinhardtii* wherein the fatty acid profile has been engineered for better fuel properties [82]. A recent study reported the production of a wide range of degree of unsaturation in TAG (designer oils) by rational modulation of type II diacyl glycerol acyltransferases in *Nannochloropsis oceanica* via reverse genetics [88].

In a typical synthetic circuit, three components are present—sensors (which sense the external inputs), processors (that process the signal and determine them) and actuators (that transmit the signal downstream which make required changes in the cell). In biological systems, regulatory circuits are mainly governed by transcriptional, post-transcriptional and post-translational components such as regulatory molecules, *cis*-elements, noncoding RNAs and transcription factors [69, 70]. In an effort to better understand the promoter structure for augmenting the nuclear gene expression, Scranton et al. [89] developed 25 synthetic algal promoters (sap) in *C. reinhardtii* by mimicking their native *cis*-elements, structure and sequences of highly expressing genes. Some of these synthetic promoters performed better than the best endogenous promoter *hsp70/rbs2*, thus opening new potentials for exploring the *C. reinhardtii* system through synthetic strategies [89]. Most of the synthetic circuits used the principle of transcriptional control that needs effective (can control the target gene expression to desired level) and programmable (can carry out functions at any desired loci) transcription factors which do not involve in cross talk.

These features ideally place newly discovered CRISPR/Cas (clustered regularly interspaced short palindromic repeats/CRISPR associated) and CRISPR–Cpf1 system as effective means to design synthetic TFs for complex synthetic gene circuits. Versatility of CRISPR/Cas system in gene regulation such as multiplex regulation (to modulate several genes simultaneously), orthogonality (ability to avoid cross talk among several regulatory modules), bi-directionality (simultaneous activation and repression of different genes) and complex activation/regulation [90, 91] using CRISPR variants has been demonstrated in several systems such as algae [14, 92], plants [93, 94], yeasts and mammals [95, 96].

#### Applications of CRISPR

CRISPR has proven to be an effective and popular strategy for exploring complex regulatory synthetic circuits [97]. In mammalian cells, synthetic biology has been demonstrated in two independent studies where the output upstream of a cascade becomes the input for a downstream module—a basic display of synthetic circuit. Here, a gRNA along with dCas9 influenced the expression of another gRNA downstream in a circuit that ultimately controls a fluorescent protein as an observable output of a final task [98, 99]. More advanced circuits such as logic gates utilizing CRISPR have also been explored in bladder cancer cells and *E. coli*. In bladder cells, dCas9 and gRNA were expressed under promoters which are cancer cell (*hTERT*) and bladder cell specific (*hUPII*). The CRISPR/dCas9 was expressed against a *lacI* repressor, whose repression relieved the expression of transgene therapeutic proteins, i.e., the output of the circuit [100]. The output of the circuit was only possible in bladder cancer cells when both promoters were active, which resembles an AND gate of an electric circuit. In *E. coli* model system, a gRNA is driven by an inducible promoter, which if expressed recruits a dCas9 to bind the constitutive promoter of a gene referred as ‘output’, thus, sterically hindering the RNA polymerase to bind and generate the output in the presence of an input signal. Output is, therefore, possible in the absence of input signal molecule, i.e., a NOT gate approach is observed [101]. The gRNA could also be expressed from two different inducible promoters each induced by different input signals. Thus, the output can be observed only when neither of the inducers are present, i.e., a NOR gate. Similarly with modifications, OR and AND gates can be constructed synthetically, thus exploring the possibility of more logical operations in a cell.

**Fig. 4:**
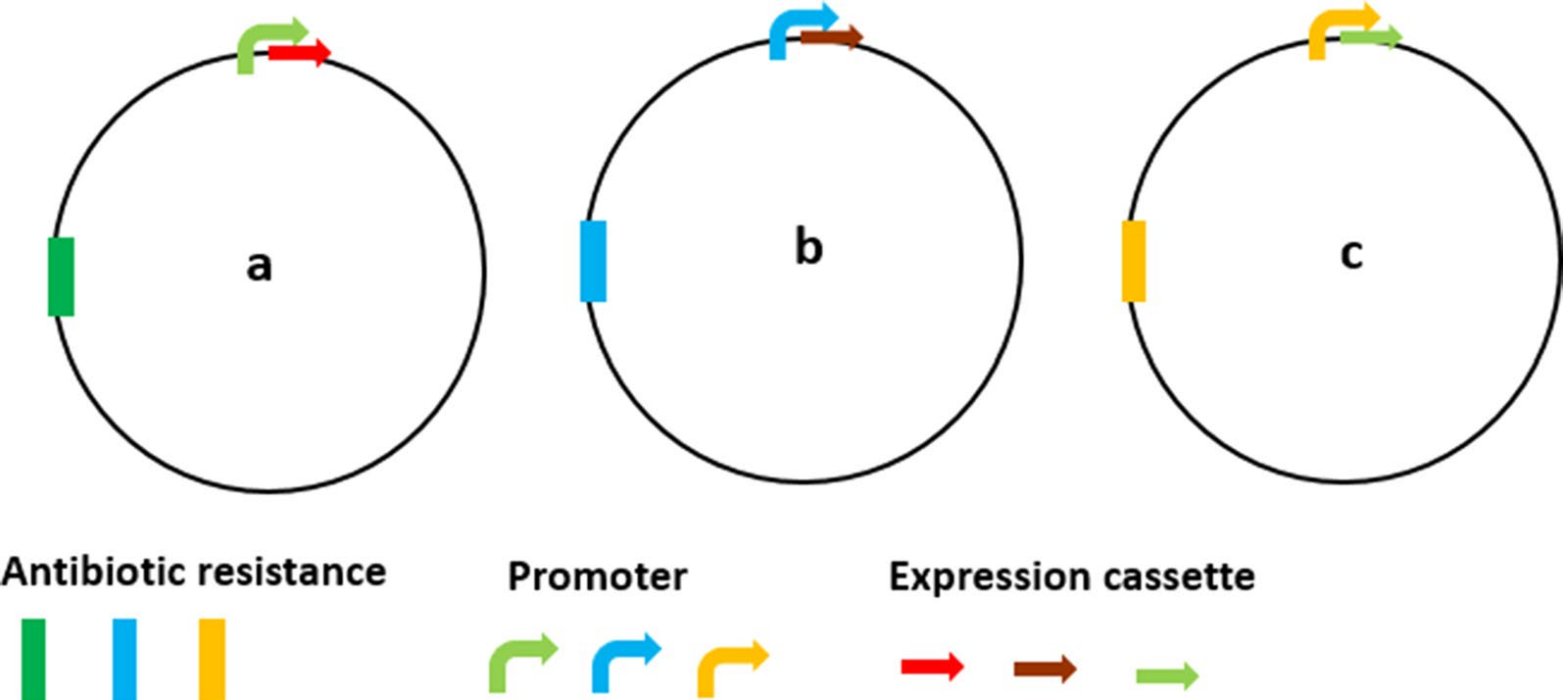
Hypothetical circuits proposed with the help of genetic modules (plasmids a, b, c) that can be applied to microalgae with light intensity stimulus. Case 1. Input (plasmid a + b) = light-inducible promoter::gRNA for transcription factor (PSR1/NRR1)^a^ + light-inducible promoter::dCas9/VP64(CRISPRa) = activation of lipid pathway (Output). Case 2. Input (plasmid a + c) = light-inducible promoter::gRNA for transcription factor (Zn(II)_2_Cys_6_)^b^ + light-inducible promoter::dCas9/SRDX(CRISPRi) = inactivation of lipid pathway suppressors (Output). ^a^PSR1 and NRR1 are transcription factors that get induced during stress which leads to lipid accumulation [171, 172]. ^b^Zn(II)_2_Cys_6_ is a transcription factor that negatively regulates lipid accumulation under nitrogen stress [183]

These concepts inspired from other systems could be utilized and attempted in microalgae and cyanobacteria through their inducible promoters/repressors, as discussed in Table 2 to build complex programmable synthetic circuits to produce products of value such as lipids, polyunsaturated fatty acids, proteins, alcohols and chemicals. Moreover, several recent studies have successfully attempted CRISPR/Cas-mediated genome engineering, enabling researchers to utilize this strategy in microalgal systems [14, 92, 102, 103]. Inducible circuits can be built in microalgae as well, with the help of inducible promoters giving inputs for driving gRNA [47, 104, 105] and light-inducible promoters activating dCas9 [106], especially because light being a known inducer of lipid synthesis helps in biofuel production (Fig. 4). A synthetic chromosome technology for microalgae was proposed by Keasling and Venter [107] for the creation of an algal platform for coordinated and controllable metabolic modifications, which would act as a designer synthetic cell committed to desired higher value product. Innovations carried out in the creation of a synthetic bacterial cell may open new horizons for microalgae synthetic biology [108]. Thus, synthetic strategies could be fully explored in various products of importance from microalgae such as lipid, bio-hydrogen, fatty acids and recombinant proteins.

**Table 2:** Promoters employed for facilitating synthetic biology in cyanobacteria and microalgae are summarized

Sl. no.	Promoters usable in synthetic biology (functional elements)	Host experimented on	Remarks about application (expressed gene, output level, significance)	References
*Promoters (metabolite induced)*				
1.	*trp*–*lac* from *E. coli* induced by IPTG (100 µM)	*Synechococcus elongatus* PCC 7942	*invA* and *glf* genes from *Zymomonas mobilis*. Increase of 160- and 30-fold for fructose (160 µM) and glucose (30 µM)	[72]
2.	*A1lacO*-*1* from *E. coli* induced by IPTG (100 mM)	*Synechocystis* sp. PCC 6803	EFE encoding gene from *Pseudomonas syringae*. Increase of eightfold (170 nL ethylene/mL h)	[152]
3.	*Trc* from *E. coli* induced by IPTG (1 mM)	*S. elongatus* PCC 7942	*uidA* gene from *E. coli.* 36-fold increase (340 nmol MU/min mg/protein)	[153]
4.	*tet* from *E. coli* induced by aTc (10^3^ ng/ml)	*Synechocystis* sp. strain ATCC27184	eYFP gene. 10,000 relative fluorescence unit. 290-fold increase	[154]
5.	(a) *Ptrc* induced by IPTG (b) Ptrc/PlacO1	*S. elongatus* PCC 7942	(a) *kviD*, *YqhD*. Isobutanol (450 mg/L). Decarboxylation of *KIV* channeled the flux towards production of isobutanol. (b) *kivd/alsS*-*ilvC*-*ilvD.* Isobutyraldehyde (1100 mg/L). Decarboxylation of *KIV* channeled towards production of metabolite	[37]
6.	*Ptrc/P*_*L*_*LacO1* induced by IPTG	*S. elongatus* PCC 7942	*ter/atoB*, *adhE2*, *crt*, *hbd.* 1-butanol (14.5 mg/L). Metabolite produced through dark anaerobic conditions	[155]
*Promoters (light inducible)*				
7.	*psbA2* from *Synechocystis* sp. PCC6803 induced by 500 μmol photons/m^2^ s	*Synechocystis* sp. PCC 6803	*ispS* gene from *Pueraria montana* 50 mg isoprene dry wt/day	[73]
8.	*psbA2* from *Synechocystis* sp. PCC 6803 induced by 50 μmol/m^2^ s	*Synechocystis* sp. PCC 6803	hydA1 gene derived from Chlamydomonas reinhardtii 130 nmol H_2_ produced mg/Chl min	[156]
9.	Endogenous CABII-1 that controls chla/b binding proteins of PSII, induced by changing light condition	Chlamydomonas reinhardtii	NIT1 nitrate reductase gene	[107]
Promoters (nutrient induced)				
10.	nirA from Synechococcus elongates PCC 7942 induced/repressed by NO_3_^−^/NH_4_^+^ with concentration 17.6 mM each	Synechocystis sp. PCC 6803	p-Hydroxyphenyl pyruvate dioxygenase gene. Increase of 25-fold (250 ng tocopherol mg/dry wt.)	[157]
11.	nirA from Synechococcus elongates PCC 7942 induced/Repressed by NO_3_^−^/NH_4_^+^ with concentration 15.0 mM/3.75 mM	S. elongates PCC 7942	cmpABCD. Increase of fivefold (260 nmol HCO_3_^−^ mg/Chl)	[158]
12.	NIT1 induced/repressed by NO_3_^−^/NH_4_^+^	C. reinhardtii, Phaeodactylum tricornutum, Dunaliella salina, Chlorella vulgaris	NIT1 nitrate reductase gene switched on/off by NO_3_^−^/NH_4_^+^. Expression vector having NIT1 promoter is used widely as circuits on expression studies	[47, 104, 105]
Promoters (metal induced)				
13.	Coat from Synechocystis sp. PCC 6803 induced by Co^2+^ (6 μM)	Synechocystis sp. PCC 6803	EFE gene from Pseudomonas Syringae. 500-fold increase (48 nL ethylene/mL h)	[152]
14.	petE from Synechocystis sp. PCC 6803 induced by Cu^2+^ (0.5 μM)	Synechocystis sp. PCC 6803	EFE gene obtained from Pseudomonas syringae. fivefold increase (28 nL ethylene/mL h)	[152]
15.	ziaA from Synechocystis sp. PCC 6803 induced by Zn^2+^ (3.5 μM)	Synechocystis sp. PCC 6803	hydA1 gene derived from Chlamydomonas reinhardtii 109 nmol of H_2_ produced mg/Chl min	[156]
16.	smt from Synechococcus elongates PCC 7002 induced by Zn^2+^ (2 μM)	Synechocystis sp. PCC 6803	EFE gene obtained from Pseudomonas syringae twofold increase (2 nL ethylene/mL h)	[152]
17.	isiAB Synechococcus sp. strain PCC 7002 repressed by Fe^3+^ (100 nM)	S. sp. strain PCC 7002	luxAB gene derived from Vibrio harveyi. twofold increase	[159]
18.	isiAB Synechocystis sp. PCC 6803 repressed by Fe^3+^ (30 μM)	Synechocystis sp. PCC 6803	isiAB+gfp genes. 5000-fold increase	[74]
19.	CYC6 endogenous promoter that controls cytochrome c6 repressed by Cu^2+^	C. reinhardtii	Inducible expression of copper responsive element (CuRe) responsive to Cu^2+^ and by Co^2+^ by CYC6	[160]
20.	Synthetic promoter sap11 produced to resemble native cis-elements and its structure	C. reinhardtii	Synthesized to understand promoter structure and increase in nuclear gene expression. Synthetic sap11 drives expression better than best endogenous promoter chimeric hsp70/rbs2. A highly conserved DNA motif was isolated by sap11 that is important for promoter function	[89]
21.	TPP riboswitch based on Thiamine pyrophosphate biosynthesis from THIC genes of Arabidopsis	C. reinhardtii	Inducible expression system depending on the presence of TPP. Conditional repression of rpoA or rps12 chloroplast genes influencing their transcription/translation	[161]
22.	RNAi silencing	C. reinhardtii	A RNAi construct effectively silenced 20 LHC proteins. 290% higher light penetration, augmentation in photosynthetic yield, less vulnerable to photo-inhibition	[162]
23.	Differential expression of rbcl mRNA maturation factor MRL1	C. reinhardtii	MRL1 under a inducible promoter can be used to regulate Rubisco level for optimum utilisation of energy according to conditions like light and CO_2_	[85]

These promoters can be utilized to construct pathways and build artificial circuits having rational, controllable logical genetic units that can yield desired output under specific stimulus

Synthetic biology not only facilitates the researchers to alter or modify metabolic pathway/s but also reprograms the cells by inserting metabolic pathways of interest. Here, the pathway of interest could be taken from one organism and inserted into another, to follow the enzymatic potential [109]. This rewiring of heterogeneous pathways into a host system leads to the creation of new metabolic networks, which induces phenotypes of interest by shifting the cell’s precedence from growth in targeted production, hence enabling construction of effective cell factories. Such insertions have been successfully performed to induce synthesis of precursors of diverse biofuel molecules, including 1-butanol [110], 2-methyl-1-butanol [111], acetone [112], ethylene [113], isoprene [73] and fatty acids [114]. The aforementioned studies clearly portray the potential role of synthetic pathways in the field of improvement of algal biofuel. The development of computational tools such as ‘pathway prediction system’ (PPS) further facilitates pathway synthesis aimed at various applications: biofuel production, degradation of xenobiotic compounds, etc. [115]. Different algorithms such as BNICE algorithm (Biochemical Network Integrated Computational Explorer) [116] and ReBiT (*R*etro-*B*iosynthesis Tool) [117] have been developed to assist in enzyme identification, thus furthering design of diverse pathways.

### Genome-scale metabolic reconstruction

Genome-scale metabolic reconstruction is an in silico technique, which utilizes biochemical models to predict microorganisms’ response to various genetic or environmental stresses. To save time and to make the method economically feasible, this method is slowly emerging as an alternate to wet lab experiments. Metabolic engineering has enabled custom designing and production of novel molecules, bearing desirable properties for wide-scale commercial, environmental and healthcare applications. Genome-scale reconstruction of an organism accurately predicts chemical transformations taking place within cellular components and focuses on delineating genetic networks governing biochemical pathways that are industrially desirable [70, 118, 119]. Classic examples of genome-scale metabolic reconstruction of *N. salina* and *Nannochloropsis gaditana* portray the importance of such in silico techniques to predict metabolic capabilities of various microalgae [120, 121]. Advances in genetics and bioinformatic tools led the way towards manipulating microalgae, envisioning improvements in light utilization, carbon flow manipulation and lipid engineering [71]. This knowledge is a powerful tool, facilitating control of strains to produce molecules of interest. With the aid of genome sequence and biochemical information, the genome-scale metabolic models (GEMs) are used to depict the absolute metabolic reactions and pathways and association between gene and protein reactions (GPR).

For the reconstruction of a novel or a less studied species, GEMs would require the entire known biochemical data and system wide data obtained through omics analyses. Progress in metabolic engineering studies and the data generated from such studies have depicted the importance of GEMs in designing new strains. Dal’Molin et al. [122] constructed a GEM (named AlgaGEM) based on the genomic sequence of *C. reinhardtii*. AlgaGEM is a complete literature-based GEM which reports the functions of 866 unique ORFs, 1862 metabolites, 2249 gene–enzyme reaction association entries and 1725 unique reactions. It was reconstructed using available compartmentalization data of cytoplasm, mitochondrion, plastid and microbody algae and compartmentalization data of *Arabidopsis thaliana.* Primarily, AlgaGEM portrays a functional metabolism for *Chlamydomonas* and anticipates distinct algal behavior. This model was validated by simulating the growth and metabolic functions obtained from the literature [122]. Chang et al. [123] reconstructed a genome-scale metabolic network for the same microalga (*C. reinhardtii*) and devised a modeling approach which predicts growth for a given light source, resolving wavelength and photon flux. They experimentally verified the transcripts, which were investigated in the network and physiologically validated model function showing high confidence in network contents and predictive applications.

A similar compartmentalized GEM was reconstructed for *Chlorella variabilis* by employing 526 genes, 1236 metabolites and 1455 reactions. This model was effective in apprehending growth of *C. variabilis* under various light conditions and facilitated analysis of metabolic pathway for the synthesis of chitosan and rhamnose. This reconstructed model will aid in providing useful information regarding target metabolites and allow improved features in the strain by metabolic engineering [124]. Yang et al. [125] proposed such a reconstruction model for a eukaryotic microalga *C. pyrenoidosa.* This model included two compartments and consisted of 61 metabolites and 67 metabolic reactions, wherein the influence of carbon and energy metabolism under various trophic modes were explained by the use of metabolic flux analysis. Metabolic reconstruction modeling combined with real-time PCR and RNA-Seq gene expression data was employed to gain an insight into mechanisms that lead to rapid TAG accumulation in *Tetraselmis* sp. M8, as the cells transitioned from a growth phase to stationary phase [126]. This study demonstrated a distinct early-stationary phase, which was distinguished by reduced cell division and increased lipid accumulation. The subsequent stationary phase was characterized by a cessation of cell division and a significant lipid accumulation. A summary of studies incorporating metabolic flux analysis has been depicted in Table 3.

**Table 3:** Recent advances in microalgae employed for the production of biofuel

Type of model or algorithm	Target product	Model-based target: knockout gene A or overexpress gene B	References
Dynamic flux balance analysis	Ethanol	Ethanol production pathway after 20 h	[163]
Genome-scale metabolic model	Prediction of growth rate with respect to photosynthetic quotient for ethanol and butanol production	Double reaction knock out of hydrolyase and dehydrogenase	[164]
In vivo carbon flux analysis	Homolanthionine	Deletion of methionine and cysteine biosynthesis repressor protein	[165]
Metabolic flux analysis	Hydrogen production	Deletion and addition of GAP1 and dehydrogenase, respectively	[166]
Metabolic flux analysis	Astaxanthin synthesis	–	[167]
Metabolic flux analysis	Fixed more CO_2_ and had a higher biomass yield	Alternate pathway for isoleucine synthesis (via citramalate synthase, CimA	[168]
Metabolic net fluxes	Hydrogen production	Disruption of hydrogenase and poly-β-hydroxybutyrate synthase	[169]

The major constraint after getting a fully annotated sequence is that it is not possible to fully predict the metabolic capabilities of the species. This leads to the construction of metabolic models, which predict probable biochemical pathways in an organism. Species-specific networks are created based on evidences from their gene–protein associations. The information about enzymes such as EC numbers, gene, protein, pathways and substrates is linked with the help of protein databases and its resources, viz. BRENDA [69] and ExPASy [25]. Visualization is an excellent approach for deeper understanding of pathways and reconstructed metabolic networks. Metabolites are represented as nodes and interactions are denoted using edges in metabolic networks. There are various web-based visualization tools for metabolic pathways, viz. BioCyc, MetaCyc, [69, 118] and Kyoto Encyclopedia of Genes and Genomes (KEGG) [17, 43, 118]. Cytoscape [77, 127, 128] is a biological network visualization and data integration tool used for visualization of the results from FBA studies [130]. CytoSEED [129] is a Cytoscape plug-in that is utilized for visualizing results from the Model SEED [69]. Fluxviz [130] is Cytoscape plug-in to visualize the flux distribution in the molecular interaction network. VANTED [131] is another data visualization and data integration tool, which could be utilized as a standalone tool. For the visualization of GEMs, a new tool has been recently developed called MetDraw [132]. This tool is compatible with systems biology markup language (SBML) file inputs and allows export of the map image as SVG files. It also grants visualization of reaction fluxes added to gene–protein expression data and metabolomics and projects all on the reconstructed network map.

### Designing predictive gene regulatory network model

Gene regulatory networks describe the complex mat of transcription factors that bind to regulatory sequences of target genes for the expression of function [133]. Gene regulatory networks (GRNs) are used to portray regulation between the genetic entities for the final expression. Computational modeling of GRNs [134] can add additional confirmation to support the wet lab findings. The sequences for transcriptional-factor-binding sites can be obtained from databases such as TRANSFAC and PROSITE, or using similarity search tools using genomic sequence. However, there is very little number of known regulatory regions. Research now focuses on developing algorithms for regulatory regions. MEME program35 is one of the popular algorithms that search for motifs in nucleotide and protein sequences that occur more often than would be predicted by chance. However, selection of the input sequence is highly sensitive.

## Optimum cultivation using response surface method and genetic algorithm

**Fig. 5:**
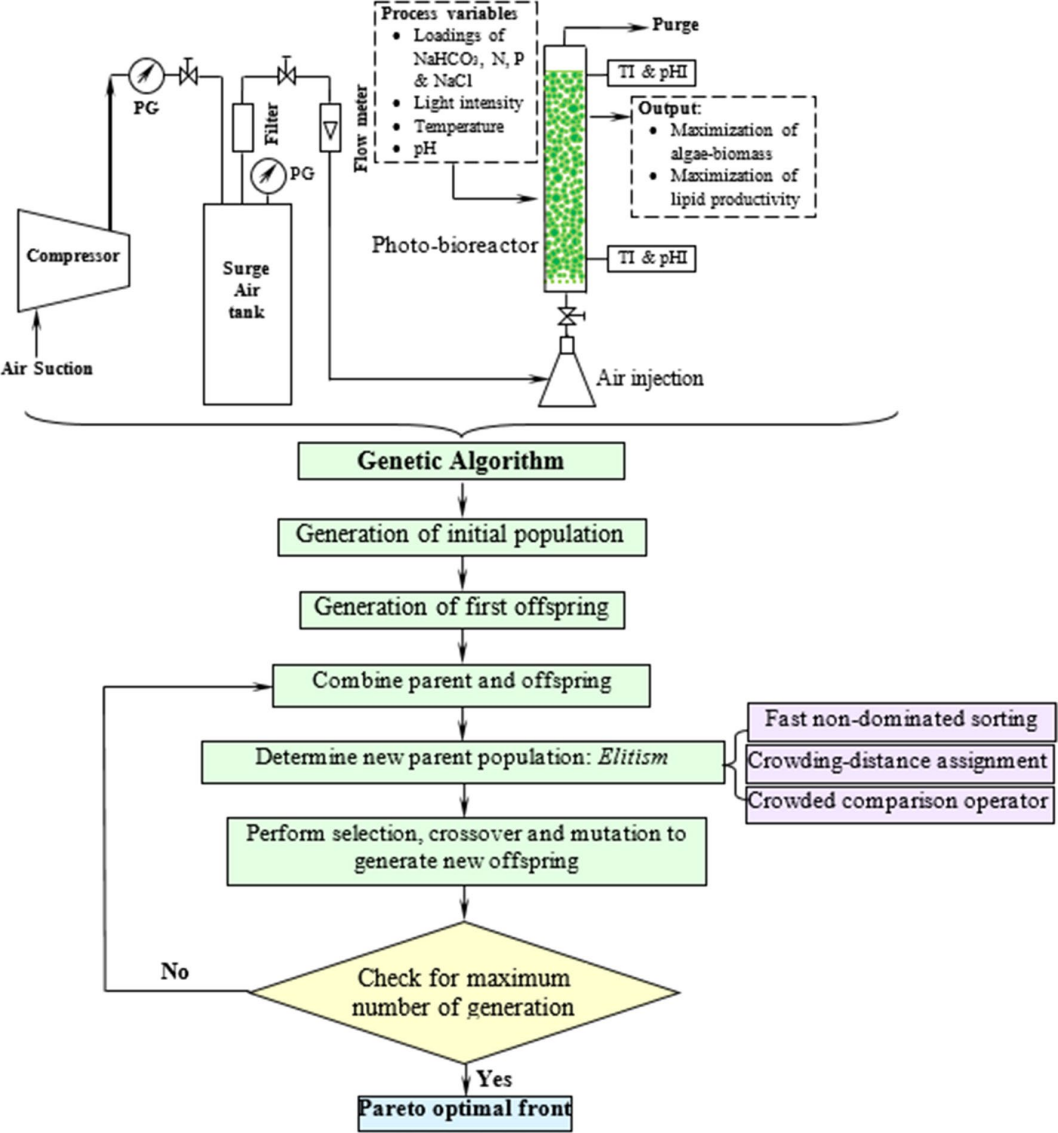
Multi-objective optimal cultivation of microalgae

There are several practical limitations in scaling up of laboratory-based algal biomass cultivation to commercial scale, economic non-feasibility being one of the major factors, as reviewed in detail by Slade and Bauen [135]. However, the cost of algal biomass can be reduced appreciably via enhanced biomass productivity and reduction in specific nutrient consumption through well-designed and optimized production system. This calls for an accelerated algal biomass production with complete recycling of the culture medium, which can be achieved by process optimization. Figure 5 summarizes multi-objective optimal cultivation strategies for microalgae. Several studies have reported improvement in algal growth through better media formulations and selecting optimum algal cultivation variables.

Algal regression models based on statistical approach and biokinetic models based on algal growth mechanism are popularly used for optimal algal cultivation. Central composite design (CCD) and response surface methodology (RSM) are tools to optimize operating conditions such as algal biomass productivity (*Y*_BM_), lipid yield (*Y*_lipid_) or CO_2_ uptake ($Y_{{{\text{CO}}_{2} }}$) from the selected design of experiments (DOEs). In general, quadratic response models are approximated to fit experimental responses in DOEs. Therefore, the responses such as*Y*_BM_, *Y*_lipid_ and $Y_{{{\text{CO}}_{2} }}$ are usually represented by second-order polynomial equations with multiple variables. The best performing algal regression model is selected on the basis of analysis of variance (ANOVA) criteria. The model with low standard deviation, high R-squared values (i.e., *R*^2^, adjusted *R*^2^ and predicted *R*^2^) and low predicted residual error sum of squares (PRESS) are selected for analysis. A gradient-based numerical search technique is employed for optimization of the selected model.

The Design-Expert software is popularly used for the development of regression model and graphical optimization. The parameters most commonly employed in algal cultivation and lipid accumulation are nutrient loading and environmental stress, including temperature, light intensity, salt and pH [136, 137]. Light intensity and the loading of nitrogen, phosphorus, potassium and their mutual interactions are the factors which primarily influence productivity in *Dunaliella tertiolecta*, whereas stress due to NaCl and light–NaCl interactions were found to significantly influence the lipid accumulation [138]. Similar effects of nutrients and environmental parameters were observed for the cultivation of *Nannochloropsis* sp., wherein algal biomass growth, lipid and eicosapentaenoic acid productivity were maximized [139]. A general observation was that enhanced lipid accumulation occurred under nutrient limitation and stressed environmental conditions. In contrast, algal growth was reduced significantly under these stressed conditions. Algal biomass and lipid productivity-involved trade-off could be handled efficiently using elitist non-dominated sorting genetic algorithm, namely NSGA-II [8, 137, 139]. Although, CCD-RSM-based model is employed to predict algal biomass productivity and lipid accumulation in algal cells; this method fails to depict the parametric effects without relating the growth kinetics. In addition, CCD-RSM model has been found to be applicable under laboratory scale only and difficult to implement under pilot/commercial scales. Therefore, one should rely on the optimum cultivation results, which are obtained on the basis of mechanistic biokinetic models for the prediction of growth behavior of microalgae.

The classical Monod/Andrews models for nutrient adsorption and Droop model involving intercellular nutrient quota are also used to predict the algal growth behavior. The rate of absorption of nutrients (e.g., N and/or P) are often influenced by the bulk concentration of nutrients (N, P, K and C) as well as NaCl concentration, which are taken care by the Monod model at the low substrate loading and Andrews inhibition model at high substrate loading.

Algal growth is also induced by the internal nutrient cell quota (i.e., N and/or P), which is described by Droop model $\left[ {{\text{i}}.{\text{e}}., \, \, \propto \left( {1 - \frac{{q_{{0,{\text{S}}}} }}{{q_{\text{S}} }}} \right)} \right]$, where *q*_0,S_ is the minimum allowable cell quota for N or P and *q*_S_ is the time variant internal quota in cell. Haldane inhibition model $\left[ { \propto \left( {\frac{{I_{\text{avg}} }}{{K_{\text{L}} + I_{\text{avg}} + \frac{{I_{\text{avg}}^{2} }}{{K_{\text{IL}} }}}}} \right)} \right]$ is commonly used to account for light-induced algal growth, where *I*_avg_ is the average algal medium light intensity, *K*_L_ and *K*_IL_ are light half-saturation and inhibition constants, respectively. Temperature-induced growth was usually taken care by incorporating Arrhenius activation $\left( {{\text{i}}.{\text{e}}., \propto {\text{e}}^{{ - \frac{{\Delta E_{\text{a}} }}{\text{RT}}}} } \right)$ and inactivation $\left( {{\text{i}}.{\text{e}}., \propto {\text{e}}^{{\frac{{\Delta E_{\text{d}} }}{\text{RT}}}} } \right)$ function in growth equation. Temperature-dependent growth could also be expressed by an empirical equation given by $\sqrt \mu = b\left( {T - T_{ \hbox{min} } } \right)\left\{ {1 - { \exp }\left[ {c\left( {T - T_{ \hbox{max} } } \right)} \right]} \right\}$ where *μ* is the specific algal growth rate, *T*_min_ and *T*_max_ are the allowable minimum and maximum temperatures for algal growth, and *b* and *c* are constants. Lee et al. [140] reviewed algal growth kinetics under different nutrient and environmental stress conditions. In a recent study, Kumar et al. proposed a detailed algal kinetics based on Monod/Andrews and Droop model to predict algal biomass growth, nutrient cell quota and lipid production [119]. Sinha et al. [141] carried out multi-objective optimization involving minimization of cultivation cost and maximization of algal biomass and lipid productivities simultaneously using NSGA-II algorithm with inheritance. It was observed that nonlinear light intensity and temperature trajectory helped to improve algal biomass productivity and cell lipid accumulation with the reduction in cultivation cost. Therefore, one can find the best possible control strategy for cost-effective accelerated biomass growth and lipid accumulation by solving multi-objective optimization problems including scale-up studies. Selection of the best combination of environmental parameters such as nutrient requirements (nitrogen, phosphorous, potassium, carbon), salinity, temperature, culture age, initial population, pH, photoperiod and light intensity significantly affects algal biomass. The response surface method and genetic algorithm are not directly related to the generation of biofuels from microalgae. However, these optimization techniques are crucial for the formulation of optimum conditions governing algal biomass growth as well as lipid production and, therefore, need to be carefully considered while designing and constructing novel microalgal systems.

## Conclusions

Microalgae bear immense potential as bio-cell factories in terms of producing key chemicals, recombinant proteins, enzymes, lipid, hydrogen, alcohol etc. Abstraction of such high-value products (algal biorefinery approach) facilitates to make microalgae-based renewable energy as an economically viable option. Synthetic biology is an emerging field that harmoniously blends science and engineering to help design and construct novel biological systems, with an aim to achieve rationally formulated objectives. The microbial genetic information, which is easily amenable to modification via metabolic engineering, systems biology and pathway reconstruction coupled with synthetic biology, allows researchers to produce required biomolecules. However, resources and tools used for such nuclear manipulation, construction of synthetic gene network and genome-scale reconstruction of microalgae are limited. The use of synthetic biology in algal biofuel production is still in its infancy, wherein challenges in the development of more advanced genetic tools, high biomass and improved CO_2_ fixation capacity need to be resolved. In addition to the aforementioned, novel consolidated bioprocess wherein a single microbe can generate renewable biofuel as an alternative to depleting fossil fuels is the need of the hour. This review also examines the role played by microalgae as biorefineries, microalgal culture conditions and various operating parameters that need to be optimized to yield biofuel that can be economically competitive with fossil fuels.

To summarize, algal biorefinery in the present state strategically produces multiple products—bulk and specialized co-product to increase the total revenue from cultivation and make the bulk production economically feasible. In practice, multiproduct large-scale biorefinery still poses several problems, which need to be soon addressed. Some economically important value-added products such as astaxanthin and oil; lutein and oil; EPA and oil are being produced in lab scale. Attempts should be made to facilitate their large-scale production through proper pathway prediction and growth and metabolic modeling, wherein the domains of systems biology and process optimization will play a crucial role. Process optimization can lead us to optimized combination of growth conditions such as light, temperature, mass transfer, reactor configuration, media formulation and supplementation, which produces improved growth kinetics. Systems biology, on the other hand, can aid in optimal use of carbon and energy balance so that every possible carbon atom is put to use and every ATP is spent judiciously taking into account the metabolism, physiology and induced stress response. This can fructify only when channeling metabolic flux and partitioning are efficient, both of which are predicted by reconstructed genome-scale metabolic models. These in silico models are based on systems biology data that stem from omics and labeling analysis.

## Abbreviations

CRISPR: clustered regularly interspaced short palindromic repeats
TALEs: transcription activator-like effectors
ZFN: zinc-finger nucleases
LEA: lipid-extracted algal
RBS: ribosome-binding sites
TAG: triacylglycerol
CRISPR/Cas: clustered regularly interspaced short palindromic repeats/CRISPR associated
PPS: pathway prediction system
BNICE: Biochemical Network Integrated Computational Explorer
ReBiT: Retro-Biosynthesis Tool
GEM: genome-scale metabolic model
GPR: gene–protein reactions
PGDB: Pathway Genome Database
ExPaSy: Expert Protein Analysis System
KEGG: Kyoto Encyclopedia of Genes and Genomes
SBML: Systems Biology Markup Language
GRNs: gene regulatory networks
CCD: central composite design
RSM: response surface methodology
DOEs: design of experiments
ANOVA: analysis of variance
PRESS: predicted residual error sum of squares
